# Absence of ultrasound inflammation in patients presenting with arthralgia rules out the development of arthritis

**DOI:** 10.1186/s13075-017-1405-y

**Published:** 2017-09-15

**Authors:** Myrthe van der Ven, M. van der Veer-Meerkerk, D. F. Ten Cate, N. Rasappu, M. R. Kok, D. Csakvari, J. M. W. Hazes, A. H. Gerards, J. J. Luime

**Affiliations:** 1 Department of Rheumatology (Na609), Erasmus MC, University Medical Centre Rotterdam, PO Box 2040, 3000 CA Rotterdam, The Netherlands; 2 Department of Rheumatology, Zuyderland Medical Centre, Heerlen, The Netherlands; 3 Department of Rheumatology, Maasstad Hospital, Rotterdam, The Netherlands; 4 Department of Rheumatology, Vlietland Hospital, Schiedam, The Netherlands

**Keywords:** Ultrasonography, Power Doppler, Arthralgia, Inflammatory arthritis

## Abstract

**Background:**

To decrease the burden of disease of rheumatoid arthritis (RA), patients at risk for RA need to be identified as early as possible, preferably when no clinically apparent synovitis can be detected. Up to now, it has been fairly difficult to identify those patients with arthralgia who develop inflammatory arthritis (IA), but recent studies using ultrasound (US) suggest that earlier detection is possible. We aimed to identify patients with arthralgia developing IA within 1 year using US to detect subclinical synovitis at first consultation.

**Methods:**

In a multi-centre cohort study, we followed patients with arthralgia with at least two painful joints of the hands, feet or shoulders without clinical synovitis over 1 year. Symptom duration was < 1 year, and symptoms were not explained by other conditions. At baseline and at 6 and 12 months, data were collected for physical examinations, laboratory values and diagnoses. At baseline, we examined 26 joints ultrasonographically (bilateral metacarpophalangeal joints 2–5, proximal interphalangeal joints 2–5, wrist and metatarsophalangeal joints 2–5). Scoring was done semi-quantitatively on greyscale (GS; 0–3) and power Doppler (PD; 0–3) images. US synovitis was defined as GS ≥ 2 and/or PD ≥ 1. IA was defined as clinical soft tissue swelling. Sensitivity and specificity were used to assess the diagnostic value of US for the development of IA. Univariate logistic regression was used to analyse the association between independent variables and the incidence of IA. For multivariate logistic regression, the strongest variables (*p* < 0.157) were selected. Missing values for independent variables were imputed.

**Results:**

A total of 196 patients were included, and 159 completed 12 months of follow-up. Thirty-one (16%) patients developed IA, of whom 59% showed US synovitis at baseline. The sensitivity and specificity of US synovitis were 59% and 68%, respectively. If no joints were positive on US, negative predictive value was 89%. In the multivariate logistic regression, age (OR 1.1), the presence of morning stiffness for > 30 minutes (OR 3.3) and PD signal (OR 3.4) were associated with incident IA.

**Conclusions:**

The presence of PD signal, morning stiffness for > 30 minutes and age at baseline were independently associated with the development of IA. Regarding the value of US in the diagnostic workup of patients with early arthralgia at risk for IA, US did perform well in ruling out IA in patients who did not have US synovitis.

## Background

Rheumatoid arthritis (RA) is a debilitating chronic autoimmune disease. Early initiation of effective disease-modifying drugs can slow disease progression and diminish joint damage [1, 2]. It could be that starting disease-modifying anti-rheumatic drug (DMARD) therapy in the arthralgia phase or even before that could lead to better patient outcomes [3, 4]. Until now, it has been fairly difficult to identify those patients with arthralgia who would benefit from such early initiation of DMARD therapy because only those who would have subsequently developed inflammatory arthritis (IA) related to a chronic inflammatory joint disease would benefit from such an early intervention. Recent technical developments in magnetic resonance imaging (MRI) and ultrasound (US) suggest that earlier detection of inflammation should be possible before clinical manifestation [5].

We know from previous research that 15% of patients with arthralgia who presented without clinical signs of inflammation at baseline would be diagnosed with IA 1 year later, half of whom were anti-citrullinated protein antibody (ACPA)-positive [6]. Although ACPA positivity is a very good predictor for those patients who will develop IA within 1 year, it is still difficult to identify the exact individuals who will develop IA, because any ACPA-positive individual has an a priori chance of 50% of developing IA. In seronegative patients, the prediction of IA is even more difficult, because only 5% develop IA within the subsequent year. Imaging techniques have been shown to be able to detect synovitis before its clinical appearance and could be of help in identifying those at risk of IA [5, 7]. MRI and US are both available in the daily rheumatology clinic. MRI has the disadvantage of being time-consuming, thereby constraining the number of joints which that can be assessed. In addition, MRI is expensive and not accessible for everyone (e.g., joint replacement, pacemaker). US is more operator-dependent because, owing to probe position, multiple examiners can make different observations. However, US is more flexible and easily applied in the clinic. In this study, we aimed to identify which patients with arthralgia will develop clinically apparent IA within 1 year using US to detect subclinical synovitis at first consultation added to demographic and clinical variables.

## Methods

This study was a multi-centre (three centres) prospective cohort study in which we followed patients with inflammatory joint complaints for 1 year.

### Patients

Patients with inflammatory joint complaints of the hands, feet or shoulders without clinically apparent synovitis in any joint were recruited from the outpatient clinic. Patients had a symptom duration < 1 year which could not be explained by other conditions, such as IA, fibromyalgia, overuse or trauma. To distinguish inflammatory arthralgia from other forms of arthralgia, patients had to have at least two painful joints in hands, feet or shoulders and two of the following criteria adapted from the Rotterdam Early Arthritis Cohort (REACH) trial [8]: morning stiffness for more than 1 h, unable to clench a fist in the morning, pain when shaking someone’s hand, pins and needles in the fingers, difficulties wearing rings or shoes, family history of RA and/or unexplained fatigue for < 1 year. Patients had to be able to understand, speak and write in Dutch. Patients received treatment as the rheumatologists saw fit, but no DMARDs were prescribed at first consultation. Written informed consent was obtained from the participants according to the Declaration of Helsinki. The study was approved by the medical ethics committee of Erasmus MC, University Medical Centre Rotterdam, Rotterdam, The Netherlands (MEC-2010-353) and was assessed for feasibility by the local ethical bodies of Maasstad Hospital and Vlietland Hospital.

### Clinical examination

A trained research nurse collected data about articular symptoms, extra-articular symptoms, family history and previous medical history. Data collection at baseline and at 6-month and 12-month follow-up included a detailed medical examination (swollen joint count in 44 joints, tender joint count in 44 joints), laboratory variables (ACPA, rheumatoid factor [RF], erythrocyte sedimentation rate), diagnosis and medications used. Observed soft tissue swelling needed to be confirmed as an arthritis by the treating rheumatologist. Because substantial loss to follow-up was expected at the start of the study due to the nature of recovering arthralgia for the majority of patients, a telephone interview was scheduled if patients did not want to return to the clinic for their 6- and 12-month evaluations. Patients were asked about their clinical symptoms. If the interviewer doubted the potential presence of clinical synovitis, patients were asked to return to the outpatient clinic for clinical evaluation.

### US examination

At baseline, trained US examiners blinded to the participants’ clinical details performed US following the European League Against Rheumatism (EULAR) guidelines concerning patient position and scanning planes [9]. To minimise inter-variability, US examiners followed a standardised scanning protocol regarding acquisition and scoring. The US machine used was the MyLab60 (Esaote, Genoa, Italy) with a high-frequency linear array probe (LA435, 10–18 MHz). Twenty-six joints were evaluated using greyscale (GS) and power Doppler (PD) imaging. We scanned metatarsophalangeal joints (MTP) 2–5 (dorsal aspect), metacarpophalangeal joints (MCP) 2–5, proximal interphalangeal joints (PIP) 2–5 (dorsal and palmar aspects) and the wrist (radiocarpal and intercarpal joints). A single midline (longitudinal 12 o’clock position) scan perpendicular to the bone surface was used as advised by the Outcome Measures in Rheumatology (OMERACT) US working group [10]. The following PD settings were used. Colour gain was set at the disappearance of colour noise. The pulse repetition frequency was set as low as possible to have maximum sensitivity but minimising noise, which resulted in a frequency of 750 Hz. We adjusted the size and position of the colour box to include the subcutaneous tissue to recognize artefacts caused by vessels above the joint [11]. PD signals were measured only in joints with a GS score ≥ 1. The total scanning time was ½ h per patient per session. The treating rheumatologist and the research nurse were blinded to the results of the US examinations at baseline.

### US evaluation

Image evaluation followed the recommendations of the Spanish Society of Rheumatology, which is a modified version of the previously developed OMERACT definitions of sonographic pathology [12]. Joints were graded according to a semi-quantitative scoring system (0–3) for both GS and PD images. For GS, all joints were graded as follows: 0 = no capsular distention; 1 = hypoechoic material only at the level of the joint margins; 2 = partial distention of the whole capsule, which appears mostly concave or flat; and 3 = complete distention of the whole capsule, which appears mostly convex. Synovial vascularisation was measured using PD and graded as follows: 0 = absent, 1 = mild single-vessel signal or isolated signal, 2 = moderate confluent vessels, and 3 = marked vessel signals in more than half of the intra-articular area [13]. US synovitis was defined as GS grade 2 or 3 and/or presence of PD (grade 1, 2 or 3).

### Outcome

One-year incident IA was defined as clinical soft tissue swelling. Observed soft tissue swelling needed to be confirmed as an arthritis by the treating rheumatologist, who was unaware of the US findings.

### Statistical analysis

If patients had no clinical evaluation for both their 6- and 12-month visits, they were classified as lost to follow-up and not included in the analysis. Simple descriptions were used to report baseline characteristics and the US findings. Depending on the distribution of the data, we used the independent *t* test or Wilcoxon-Mann-Whitney test to examine differences between cases and non-cases. Frequencies were compared using a chi-square test. Sensitivity and specificity were used to assess the diagnostic value of US for the development of IA.

After consideration of the available literature [14, 15], we identified the following variables as relevant in the association with emerging IA: demographic characteristics (age, sex), clinical characteristics (tender joint count, high positive autoantibodies [ACPA, RF], morning stiffness lasting ≥ 30 minutes) and US findings (presence of US synovitis, positive PD signal in at least one joint) [14–16]. These variables were tested for their association with IA using univariate logistic regression. For multivariate logistic regression, we used a backward stepwise model procedure to select the strongest predictors (*p* = 0.157) [17]. A *p* value of 0.157 is equal to the Akaike information criterion for predictors with one regression coefficient and is recommended for use in stepwise selection of predictors [18]. Missing values of independent variables were handled by multiple imputation using the STATA multiple imputation by chained equations routine (*M* = 20) [19]. Analysis was done using STATA 14 software (StataCorp, College Station, TX, USA).

## Results

**Fig. 1:**
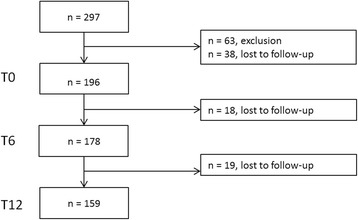
Flowchart showing the distribution of patients during follow-up

In total, 297 patients were recruited to participate. The flowchart in Fig. 1 shows the distribution of patients during follow-up. At baseline, 196 patients met the inclusion criteria. One hundred seventy-eight patients (91%) returned for their clinical evaluation at 6 months, and 159 patients (81%) had their 12-month assessment. We could determine our primary outcome for 174 patients (89%).

In total, 31 (16%) patients had developed IA within 1 year of follow-up, of whom 15 had started DMARD therapy. Twenty-two patients had no definite diagnosis: 12 patients had mono-arthritis, and 10 patients had poly-arthritis. A definite diagnosis after 12 months was given for nine patients (RA: *n* = 4; psoriatic arthritis: *n* = 4; spondyloarthritis: *n* = 1). Baseline characteristics of patients with IA and non-IA patients are shown in Table 1. We found a statistically significant difference in baseline characteristics between the IA and non-IA groups for age (mean 50 vs 44 years; *p* = 0.005). In addition, US synovitis was found more often in patients with IA than in non-IA subjects (59% vs 32%; *p* = 0.007), and PD signal was present in 31% of the patients with IA vs 12% of the non-IA patients (*p* = 0.012).

**Table 1:** Baseline characteristics (*n* = 174)

	Patients with IA (*n* = 31)	Non-IA patients (*n* = 143)	*p* Value^a^
Female sex, *n* (%)	25 (81)	119 (83)	0.731
Age, years, mean (SD)	50 (8)	44 (12)	0.005
BMI, mean (SD)	26.8 (4.4)	27.5 (5.2)	0.534
SJC44, median (IQR)	0 (0–0)	0 (0–0)	–
TJC44, median (IQR)	4 (2–9)	5 (3–8)	0.828
RF-positive, *n* (%)	9 (31)	37 (27)	0.628
ACPA-positive, *n* (%)	7 (24)	19 (14)	0.161
ESR, median (IQR)	10.5 (5–22)	10.5 (5–21)	0.824
Morning stiffness, minutes, median (IQR)	30 (30–60)	30 (15–60)	0.515
DAS28, mean (SD)	3.4 (1.1)	3.3 (1.0)	0.710
US synovitis^b^, *n* (%)	17 (59)	44 (32)	0.007
PD score > 0, *n* (%)	9 (31)	17 (12)	0.012

*Abbreviations: ACPA* Anti-citrullinated protein antibody, *BMI* Body mass index, *DAS28* Disease Activity Score in 28 joints, *ESR* Erythrocyte sedimentation rate, *IA* Inflammatory arthritis, *PD* Power Doppler, *RF* Rheumatoid factor, *SJC44* Swollen joint count in 44 joints, *TJC44* Tender joint count in 44 joints, *US* Ultrasound

^a^ Depending on the distribution of the data, we used the independent *t* test or the Wilcoxon-Mann-Whitney test. Frequencies were compared using a chi-square test (*p* ≤ 0.05)

^b^ US synovitis defined as greyscale grade 2 or 3 and/or presence of PD (≥1)

### US findings

US findings are described in more detail in Table 2. In total, 72 patients with arthralgia (37%) had US synovitis, of whom 29 had a positive PD signal. Wrists (26%) and MTP (11%) were most commonly involved, which was also observed if only PD was taken into consideration. The distribution of US synovitis over the different joint groups between patients who developed IA and those who did not develop IA was comparable, except for the MTP, which were more involved in the IA group.

**Table 2:** Distribution of ultrasound findings

	US synovitis^a^, *n* (%)		PD-positive, *n* (%)	
	IA (*n* = 31)	Non-IA (*n* = 143)	IA (*n* = 31)	Non-IA (*n* = 143)
US-positive	17 (55)	45 (31)	9 (29)	17 (12)
MCP	3 (10)	9 (6)	1 (3)	3 (2)
PIP	3 (10)	1 (1)	2 (6)	0 (0)
Wrists	8 (26)	35 (24)	4 (13)	15 (10)
MTP	9 (29)	11 (8)	4 (13)	2 (1)

*Abbreviations: IA* Inflammatory arthritis, *MCP* Metacarpophalangeal joint, *MTP* Metatarsophalangeal joint, *PD* Power Doppler, *PIP* Proximal interphalangeal joint, *US* Ultrasound

^a^US synovitis defined as greyscale grade 2 or 3 and/or presence of PD (≥ 1)

### Diagnostic value of US

The sensitivity and specificity of US synovitis in relation to the incidence of IA if one joint was positive on US were 59% and 68%, respectively. Positive predictive value (PPV) was 26%, and negative predictive value (NPV) was 74%. When we required two joints to be US-positive to identify an IA case, sensitivity decreased to 28% and specificity increased to 86% (PPV 27%, NPV 73%). For the presence of PD signal, sensitivity was 31% and specificity was 88% for one positive PD joint (PPV 33%, NPV 67%). When two joints were required, sensitivity decreased to 14% and specificity increased to 95% (PPV 38%, NPV 63%). If no joints were positive on US, the NPV was 89%.

### Association of independent variables with development of IA

To quantify the associations between baseline characteristics and incident IA at follow-up, we performed univariate and multivariate logistic regression after multiple imputation (*M* = 20). Results are presented in Table 3. Age (OR 1.06, 95% CI 1.03–1.09), morning stiffness > 30 minutes (OR 2.39, 95% CI 1.20–4.73) and positive ACPA (OR 2.08, 95% CI 1.07–4.07) were associated with IA in univariate analysis. Other clinical and demographic characteristics did not differentiate patients with IA from non-IA patients. For the presence of US synovitis in at least one joint, the OR was 3.03 (95% CI 1.69–5.41), and for the presence of PD signal in at least one joint, the OR was 3.12 (95% CI 1.61–6.03). In the multivariate logistic regression analysis, age (OR 1.06, 95% CI 1.03–1.10), morning stiffness > 30 minutes (OR 2.80, 95% CI 1.33–5.90), positive ACPA (OR 2.35, 95% CI 1.13–4.87) and US synovitis (OR 2.65, 95% CI 1.44–4.88) remained associated with the development of arthritis during 1 year of follow-up. If we replaced US synovitis with the presence of PD signal (OR 3.44, 95% CI 1.71–6.95), the ORs for age and morning stiffness were similar, but positive ACPA was not associated with the development of arthritis.

**Table 3:** Association between baseline characteristics and development of inflammatory arthritis using univariate logistic regression analysis and multivariate logistic regression analysis after multiple imputation (*n* = 174)

	Univariate model		Multivariate model including US synovitis		Multivariate model including presence of PD	
	OR (95% CI)	*p* Value	OR (95% CI)	*p* Value	OR (95% CI)	*p* Value
Demographics						
Age, years	1.06 (1.03–1.09)	< 0.001	1.06 (1.03–1.10)	< 0.001	1.07 (1.04–1.10)	< 0.001
Sex	0.84 (0.42–1.70)	0.627				
BMI	0.98 (0.92–1.04)	0.438				
Clinical variables						
Tender joints, range 0–44	1.01 (0.96–1.07)	0.676				
DAS28	1.21 (0.92–1.58)	0.175				
Morning stiffness > 30 minutes	2.39 (1.20–4.73)	0.013	2.80 (1.33–5.90)	0.007	3.34 (1.60–6.96)	0.001
Rheumatoid factor-positive	1.21 (0.65–2.23)	0.545				
ACPA-positive	2.08 (1.07–4.07)	0.032	2.35 (1.13–4.87)	0.021		
ESR	1.00 (0.98–1.02)	0.850				
US^a^						
US-positive	3.03 (1.69–5.41)	< 0.001	2.65 (1.44–4.88)	0.007		
PD-positive	3.12 (1.61–6.03)	0.001			3.44 (1.71–6.95)	0.001

*Abbreviations: ACPA* Anti-citrullinated protein antibody, *BMI* Body mass index, *DAS28* Disease Activity Score in 28 joints, *ESR* Erythrocyte sedimentation rate, *IA* Inflammatory arthritis (defined as clinical soft tissue swelling), *PD* Power Doppler, *US* Ultrasound

^a^US synovitis definition: greyscale > 1 and/or PD > 0

## Discussion

Sixteen percent of patients with early arthralgia developed IA after 1 year of follow-up, of whom 59% showed US synovitis at baseline. Age, morning stiffness > 30 minutes and positive PD signal were all significantly associated with the development of IA after 1 year in a multivariate model. Regarding the value of US in the diagnostic workup of patients with early arthralgia at risk for IA, US did not perform well in ruling in IA (PPV 26%), but it did perform well in ruling out IA in patients who did not have US synovitis (NPV 89%).

Up to now, researchers in few studies had investigated subclinical synovitis in patients with arthralgia by making use of imaging modalities. In an autoantibody-positive arthralgia cohort, patients with positive US had an increased risk for IA [14, 15]. This was confirmed in our study, although only 15% of the patients were ACPA-positive, and 24% was RF-positive. In another study in which investigators evaluated patients with very early hand symptoms, the presence of PD signal was associated with IA in addition to clinical features (e.g., swollen joints) and laboratory tests (e.g., serology, RF, ACPA) [20]. For MRI, the results are not conclusive. Among a seropositive arthralgia population, changes on MRI indicative of MCP and PIP inflammation were not associated with the development of arthritis at 3-year follow-up [21]. In contrast, MRI findings in the most affected hand in patients with clinically suspect arthralgia showed that subclinical MRI inflammation preceded clinical arthritis by a few months. This was also found in a subanalysis in a seronegative arthralgia population [22, 23].

Our results should be interpreted in the light of the choices we made. As explained in the Background section above, we aimed for very early identification of IA. For this study, we restricted the population to patients with at least two painful joints as well as two criteria related to inflammation to be surer of the inflammatory component. These inclusion criteria may have driven the selection to a population at increased risk for polyarthritis. We missed those patients who might be at risk for IA but had only one painful (large) joint. However, our inclusion criteria are in line with other arthralgia cohorts and with the new EULAR guidelines regarding clinically suspect arthralgia [22, 24]. Other forms of selection may have occurred due to rheumatologists who recruited clinically suspected patients with possibly more severe symptoms [25]. In addition, 38 patients decided not to participate without giving specific reasons, which could have introduced a bias toward patients with more severe complaints. These patients did not differ in age and sex compared with the responders, but we do not know whether their clinical symptoms differed. Information bias could have occurred because some patients were lost to follow-up (14%). This was anticipated at the start of the study, so we included a telephone service if patients did not respond to their initial invitation for follow-up. If those patients did not wish to return, they were asked a small set of questions to establish whether they were at risk of IA. We saw no differences in their baseline characteristics compared with those returning to the clinic. We did not include these patients in the analysis. Other bias could have been introduced by not blinding the clinical examination and US examination, because we included only patients with arthralgia. This could have led unconsciously to less sensitive assessment of clinical and US synovitis. However, at baseline, several patients were excluded because of clinically apparent arthritis confirmed by a trained research nurse. Another item to take into account is that US is still considered operator-dependent; therefore, the US examiners scanned patients following the US study protocol as training prior to the start of the study. In addition, US examiners followed a protocol regarding acquisition and scoring. Previous research regarding inter-reliability confirmed that a consensus scoring system combined with a standardised acquisition protocol provided good inter-reliability [26, 27]. In our definition of US synovitis, we combined GS abnormalities with PD signal. Studies showed that GS abnormalities also occur in non-arthritic individuals, and especially the discriminative value of GS score 1 is debatable [14, 28]. Therefore, we used a threshold score of 2 for GS US abnormalities.

## Conclusions

Sixteen percent of the patients with arthralgia developed IA after 1 year of follow-up, of whom 59% showed US synovitis at baseline. Positive PD signal, morning stiffness and age were independently associated with the development of IA after 1 year. Given the high NPV, US has added value to identify which patients would not develop into IA. Further research is recommended to confirm our results regarding the diagnostic value of US synovitis to predict the progression to IA in patients with early arthralgia.

## Abbreviations

ACPA: Anti-citrullinated protein antibody
BMI: Body mass index
DAS28: Disease Activity Score in 28 joints
DMARD: Disease-modifying anti-rheumatic drug
ESR: Erythrocyte sedimentation rate
EULAR: European League Against Rheumatism
GS: Greyscale
IA: Inflammatory arthritis
MCP: Metacarpophalangeal joint
MTP: Metatarsophalangeal joint
MRI: Magnetic resonance imaging
NPV: Negative predictive value
OMERACT: Outcome Measures in Rheumatology
PD: Power Doppler
PIP: Proximal interphalangeal joint
PPV: Positive predictive value
RF: Rheumatoid factor
SJC: Swollen joint count
TJC: Tender joint count
US: Ultrasound
